# Magnitude and Factors Associated with Refer Results of Newborn Hearing Screening at Academic Tertiary Level Hospital, Addis Ababa, Ethiopia

**DOI:** 10.1155/2022/1977184

**Published:** 2022-07-07

**Authors:** Haben Birhane Werkineh, Uta fröschl, Wale Limeneh Gellaw, Fantu Lombamo Untiso, Lense Gelaneh Negash

**Affiliations:** ^1^Department of ENT, Hawassa University Comprehensive Specialized Hospital, Hawassa, Ethiopia; ^2^Department of ENT, Otology-Unit, St. Paul's Hospital Millennium Medical College, Addis Ababa, Ethiopia; ^3^Department of ENT, Pediatric Otolaryngology-Unit, St. Paul's Hospital Millennium Medical College, Addis Ababa, Ethiopia; ^4^School of Public Health, St. Paul's Hospital Millennium Medical College, Addis Ababa, Ethiopia; ^5^Department of ENT, St. Paul's Hospital Millennium Medical College, Addis Ababa, Ethiopia

## Abstract

**Background:**

Hearing impairment is a leading cause of disability worldwide. Early identification and early intervention of hearing loss can prevent further disability in the development of speech, language, cognition, and other developmental domains. This study aimed to determine the magnitude and factors associated with the refer results of newborn hearing screening at an academic tertiary hospital.

**Methods:**

An institution-based time series cross-sectional study was conducted with a calculated sample size of 368 newborns selected by systemic random sampling from a total of 2087 newborns born in SPHMMC during the study period. Two stage screening protocol was conducted using Transient Evoked Otoacoustic Emission (TEOAE) on the first, followed by TEOAE and Auditory Brainstem Reflex (ABR) as a second stage for newborns with refer results. Data were analyzed using Statistical Package for Social Sciences (SPSS) version 26.0. Bivariate and multivariate analyses between dependent and independent variables were performed using binary logistic regression with a significance level of *P* value <0.05.

**Result:**

Of the total sample size of 368 newborns, 62% (228) passed the first TEOAE and 38% (140) yielded refer results. From 121 who came for follow-up screening (6–28 days), 49.5% (60) passed the second TEOAE and 50.5% (61) had refer results. AABR screening of 61 newborns yielded pass in 11.5% (7) and refer result in 88.5% (54) newborns. Noise (AOR= 4.746, 95% CI 2.505–8.992, *P* < 0.001), vernix caseosa (AOR= 19.745, 95% CI 9.057–43.043, *P* < 0.001), and very low birth weight (AOR= 4.338, 95% CI 1.338–14.067, *P*=0.015) were found to be significantly associated with the refer rate of the first TEOAE test. Noise (AOR 39.445, 95% CI 5.974–260.467, *P* < 0.001) and neonatal jaundice (AOR 21.633, 95% CI 1.540–303.994, *P*=0.023) were significantly associated with the follow-up screening refer result of TEOAE. Repeat TEOAE has decreased the refer rate from 38.0% (140/368) to 17.5% (61/349), 19 of which were lost to follow-up. AABR has decreased the overall refer rate from 17.5% to 15.5% (54/349).

**Conclusion:**

This study shows a significant number of newborns (15.5%) who need diagnostic audiologic work-up and may need intervention. Vernix caseosa and noise are avoidable factors, but newborns with jaundice and very low birth weight should be sent to ENT for screening.

## 1. Introduction

Hearing impairment is a significant cause of disability worldwide, and more than two-thirds of the population with hearing impairment live in developing countries [1–3]. Approximately 15% of the world's adult population has some degree of hearing loss. Over 5% of the world's population or 466 million people are estimated to be living with disabling hearing loss, and out of those, 34 million are children. If the current trend continues, it is estimated that by 2050, over 900 million people will have a hearing impairment. According to the World Health Organization (WHO), 60% of childhood hearing impairment is preventable [3].

Hearing loss appears to be more common in sub-Saharan Africa than the developed parts of the world. The WHO estimates the prevalence of hearing loss (defined as hearing loss >35 dB) for adults aged >15 years old which was 15.7% in sub-Saharan Africa vs. 4.9% in high-income countries. For children aged between 5 and 14 years, the prevalence was estimated at 1.9% in sub-Saharan Africa vs. 0.4% in high-income countries. However, the estimates are based on a very limited evidence base, all of which relied on school-based hearing screening [1–5].

Early detection of hearing loss is conducted through newborn hearing screening (NHS). Screening protocols and measures used within NHS programs worldwide differ. For example, screening protocols in India consist of three stages with TEOAE at the first and second stages of screening followed by AABR at the third stage. In comparison, hospitals in the United States employ a two-stage screening protocol with TEOAE and AABR screening at both stages [4].

There are 2 approaches to screening newborns for hearing loss: universal newborn hearing screening (UNHS) of all newborns and targeted screening (TNHS) of high-risk newborns [6].

The optimal approach for implementing infant hearing screening remains a subject of discussion, but the Joint Committee on Infant Hearing (JCIH) of the USA has consistently since year 2000 recommended targeted newborn hearing screening (TNHS) for developing countries based implicitly on its list of risk factors [7]. These risk factors include family history of early or progressive childhood hearing loss, neonatal sepsis, neonatal jaundice, neonatal intensive care unit admission for more than 5 days, aminoglycoside administration for more than 5 days, perinatal asphyxia, prematurity <34 weeks, very low birth weight <1500 grams, and meningitis/encephalitis [7].

The presence of hearing impairment in a newborn baby may result in devastating long-term consequences. These include communication delays, emotional disturbances, cognitive deficits, and, subsequently, future employment difficulties and career limitations. It also has an economic impact on the family since the societal cost for providing education and health services is high. Failure to make the diagnosis within the first 6 months of life results in the failure to achieve vital stages in speech and language development and consequently indicates a poorer prognosis for the individual regarding their cognitive abilities. It is therefore imperative to detect hearing impairment as early as possible in neonates and infants [2, 8–10].

The US JCIH recommends 1-3-6 benchmark (screening completed by 1 month, audiologic diagnosis by 3 months, and enrollment in early intervention by 6 months), but one should strive to meet a 1-2-3 month timeline. The earliest possible age of identification is encouraged for two reasons. First, the infant can receive earlier intervention for auditory and/or visual access to the language. Second, objective audiologic testing can be completed without sedation during the natural sleep that occurs when newborns/infants are young enough to sleep for prolonged periods of time [10–15].

One way of preventing hearing impairment and its impact is by early identification and intervention through newborn hearing screening (NHS). However, we don't have NHS programs in our set-up. This study aims to determine the magnitude of the newborn refer results who need diagnostic audiologic work-up and hence link them to the ENT department for early identification as well as intervention. However, it needs integrated interdepartmental work between Obstetrics, Neonatology, and ENT departments. Refer rates varies from study to study depending on the protocols and measures (TEOAE, AABR) used. A systematic review was done in Canada 2012 on OAE`s in newborn hearing screening and the effects of different protocols on test outcomes which included ten articles, with a total of 119,714 newborn participants. The pooled referral rate was 5.5%. Individual referral rates ranged from 1.3% to 39.7% [5].

Most studies indicated rescreening, and increasing age at initial screening has reduced the refer rate as well as high false positive rate [16–23]. The overall refer rates of NHS programs in developing and developed countries vary considerably. The refer rate of developing countries such as Ghana (16.9%), Colombia (15.9%), South Africa (16.7%), and Nicaragua (30.8%) was higher compared to Western countries such as the United Kingdom (2.6%), Italy (3.8%), Germany (5.5%), Belgium (2.8%), France (1.5%), USA (1.6%), and Brazil (0.2%) [24].

In general, most literature works and guidelines have stressed the importance of NHS programs. The WHO also recommends the implementation of NHS programs for early detection and intervention of hearing loss. It has been noted that most developed countries are implementing UNHS compared to TNHS. The use of different screening protocols and stages has been described, with the use of OAE and ABR being the most common screening tests. AABR has a lower refer rate compared to TEOAs as suggested by previous studies [24].

This study aims to show the significance of newborn hearing screening in our setup as well as an estimate of newborns at risk of hearing loss because of the lack of the newborn hearing screening program in our setup. Therefore, this study will be used to develop NHS programs and guidelines for UNHS, targeted newborn screening (high risk), as well as early hearing detection and intervention to perform a diagnostic audiologic work-up in newborns with refer results. Furthermore, the findings are helpful to address the burden and impact of childhood hearing impairment in resource-limited setups as in our country and elsewhere.

## 2. Methods and Materials

### 2.1. Study Setting

The study was conducted in the postpartum ward, labor ward, and NICU of St Paul's Hospital Millennium Medical College (SPHMMC) which was established in 1968. It is a rapidly expanding comprehensive specialized hospital especially after it started the medical college in 2000 E.C/2007G.C. Currently, it has 19 departments with 14 general specialty training and 16 subspeciality training departments. A hospital-based time series cross-sectional study was conducted on newborns born in SPHMMC from June 16 to August 31, 2021, GC. All neonates born during the study period and younger than 28 days were eligible. Based on previously published [5] literature, a population proportion (*P*) of 39.7% refer rate was used, and hence, our final sample size was 368. From a total of 2087 newborns born in SPHMMC during the study period fulfilling the eligibility criteria, 368 patients were selected using systematic random sampling (*K*= 4) and were included in this study.

### 2.2. Data Collection Instruments and Screening Tools

The data were collected using a prepared semi-structured questionnaire with components of socio-demographic characteristics and risk factors of hearing loss. All parents of infants to be screened were provided with an information brochure prior to screening. Screening was conducted in the labor ward, postpartum ward, neonatology unit, and audiology unit (for follow-up screening). After informed consent was obtained from a parent, each newborn was screened. Two-stage screening was performed, first using TEOAE at several points in time as early as possible after birth.

**Figure 1:**
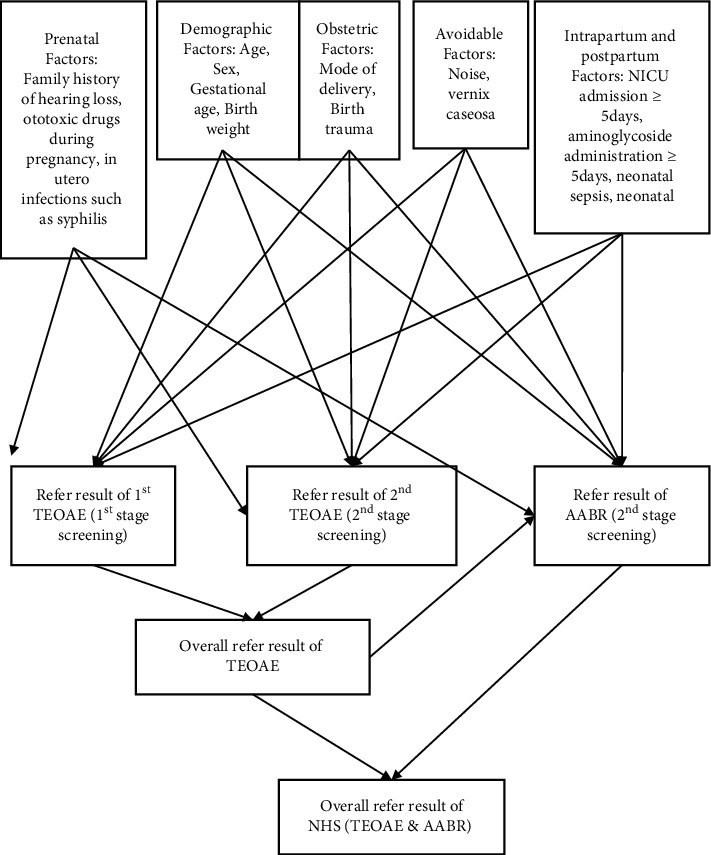
Data collection tool includes semi-structured questionnaire which includes demographic, prenatal, obstetric, intrapartum, and postpartum factors. Screening stages and its process (4).

Second stage screening was done in ears that yielded a unilateral or bilateral refer result within 4 weeks using TEOAE, followed by ABR (Figure 1). Too noisy was defined by the noise parameters set on the machine. SENTIERO ADVANCED (serial number 3300186 and calibration date 2019-10-01) was used, which offers conventional and speech audiometry, tympanometry, OAE, and ABR in one device. TEOAE screening tests frequencies of 1 kHz, 1.5 kHz, 2 kHz, 3 kHz, 4 kHz, 5 kHz, 6 kHz, and 8 kHz using a nonlinear click stimulus level of 80 dB SPL peak. Results will be either pass if 8 out of 8 frequencies have a valid response or refer if the valid response is <8 out of 8 frequencies. AABR results will be either pass if there is a valid response to a click stimulus level of 35 dB eHL or refer if there is no valid response to the stimulus level of 35 dB eHL. Records of the study population were assessed for completeness before data entry.

## 3. Results

### 3.1. Demographic Characteristics

**Figure 2:**
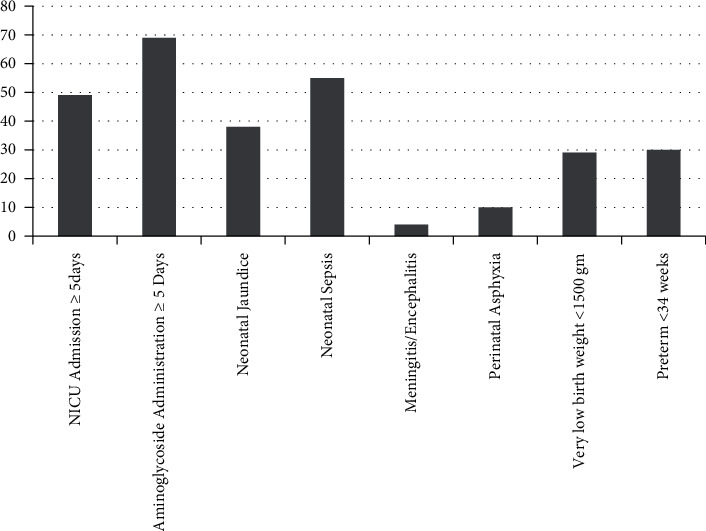
Bar chart frequency of risk factor assessment of newborns (5).

In our study, 368 newborns were screened. Out of them, 172 (46.7%) were females and 196 (53.3%) were males. Age in days was 1–30 days with 212 (57.6%) were ≤24 hours old, 103 (28.0%) were >24 hrs–7 days old, 53 (14.4%) were >7–28 days old. The median age was 1 day. 30 (8.2%) newborns were <34 weeks of gestational age with 338 (91.8%) were ≥34 weeks of gestation (Figure 2). 262 were of normal birth weight [2500–4000 grams], 77 were of low birth weight [1500–2500 grams), and 29 were of very low birth weight (<1500 grams).

### 3.2. Magnitude of Postnatal, Prenatal, and Obstetric Related Factors

High ambient noise (intensity 50–65 dB) was observed in the neonatology unit and labor ward, but the postpartum ward was very quiet. 201 (54.6%) newborns were screened in the postpartum wards, 57 (15.5%) in the labor ward, and 110 (29.9%) in the neonatology unit. All mothers were not screened for toxoplasmosis, rubella, CMV, and herpes simplex virus infection because these investigations are highly costly and not readily available.

On physical examination, 63 (17.1%) newborns had vernix caseosa, 3 (0.8%) had preuricular tags, and 1 newborn had grade 1 microtia. 5 newborns were diagnosed to have down syndrome, and one had spina bifida. Postnatal risk factors of hearing loss, such as neonatal sepsis and aminoglycoside administration for ≥5 days, were found in 55 (14.9%) and 69 (18.8%) newborns, respectively (Figure 2).

### 3.3. First Screening Outcome of TEOAE

Out of all our participants, 228 newborns (62%) passed the first TEOAE screening test, and 140 newborns (38%) yielded the refer results. The refer rate was 37.3% (79/212) in the age group tested within ≤24 hours, 32% (33/103) tested within >24 hours–7 days, and 52.8% (28/53) in neonates >7–30 days. Newborns tested ≤24 hours after delivery and between >24–7 days have similar refer rates, but those newborns tested >7–28 days of age had higher refer rates.

The referral rate in the noisy environment was 57.8% (96/167), compared to 21.8% (44/201) in the quiet environment. The refer rate in newborns with vernix caseosa was 77.8% (49/63) compared to those without in 29.8% (91/305) of newborns. The high refer rate was found in newborns with neonatal sepsis, jaundice, NICU admission, and aminoglycoside administration (Table 1).

110 newborns were screened in the neonatology unit, and 258 were in the postpartum ward and labor ward. The refer rate in the neonatology unit was 59.9% (65/110) compared to the postpartum and labor ward which was 29.1% (75/258). A higher refer rate was observed in the neonatology unit during the first TEOAE screening.

On binary regression analysis, the following factors were found to have a significant association (*P* value <0.05) with the first screening outcome; age, noise, vernix caseosa, gestational age, mode of delivery, birth weight, neonatal jaundice, neonatal sepsis, NICU admission >5 days, screening site, aminoglycoside administration >5 days, and perinatal asphyxia (Table 2).

On multivariable logistic regression analysis, noise (AOR= 4.746, 95% CI 2.505–8.992, *P* < 0.001), vernix caseosa (AOR= 19.745, 95% CI 9.057–43.043, *P* < 0.001), and very low birth weight (AOR= 4.338, 95% CI 1.138–12.395, *P*=0.015) were found to be significantly associated with the refer rate of the first screening outcome of TEOAE (Table 2).

### 3.4. Follow-Up Screening Outcome

Follow-up screening was done in individuals with refer results of the first TEOAE screening test. In our follow-up screening, the TEOAE test was used first, followed by AABR for those who failed the second TEOAE test. Out of the 140 newborns who failed the initial screening, 121 newborns came for follow-up screening, and 19 were lost to follow-up. Out of the 121 newborns screened for follow-up using TEOAE, 60 (49.6%) passed and 61 (50.4%) had refer results. The age of newborns enrolled for follow-up screening was as follows: 3 (2.5%) newborns were 2 days old, 74 (61.2%) were >2 days–7 days old, and 44 (36.4) were >7–28 days.

Follow-up screening was done in the neonatology unit, postpartum ward, and ENT meeting room. Out of 121, 41 (33.9%) were screened in a noisy environment (Neonatology unit) and 80 (66.1%) were in a quiet environment (Postpartum ward and ENT meeting room). The refer rate in female and male newborns was 43.6% (24/55) and 56.0% (37/66), respectively. There was a high refer rate in newborns with very low birth weight and the preterm was ≤34 weeks of gestation (Table 3).

The refer rate was high in newborns with neonatal jaundice, sepsis, aminoglycoside administration >5 days, NICU admission >5 days, asphyxia, and those screened in a noisy environment as well as age during the second screening test. From these factors: neonatal jaundice, aminoglycoside administration >5 days, NICU admission >5 days, noise, birth weight, and age were found to be significantly associated with the refer rate of the second TEOAE screening test (*P* value <0.05) on bivariate regression analysis.

However, only noise (AOR 39.445, 95% CI 5.974–260.467, *P* < 0.001) and neonatal jaundice (AOR 21.633 95% CI 1.540–303.994, *P*=0.023) were significantly associated with the second screening refer rate of the TEOAE test on multivariate regression analysis (Table 4). Second, screening using TEOAE has decreased the refer rate from 38.0% (140/368) to 17.5% (61/349, 19 lost to follow-up).

### 3.5. Follow-Up Screening Outcome of ABR

ABR screening was conducted on 61 of those who failed the second TEOAE test. From 61 newborns, 7 (11.5%) passed and 54 (88.5%) had still a refer result. 54 of the newborns were linked to the ENT department for diagnostic audiologic work-up. ABR screening has decreased the refer rate to 15.5% (54/349). In general, 295 (80.1%) passed our screening test and 54 (14.7%) had refer results and 19 (5.2%) were lost to follow-up.

Age and noise at second screening were found to be significantly associated with the AABR screening outcome on binary regression analysis with CI of 95% (*P* values-0.002 and 0.018, respectively). However, there was no significantly associated factor on multivariable logistic regression analysis.

## 4. Discussion

We have screened 110 (29.9%) newborns from the Neonatology unit, 201 (54.6%) from the postpartum ward, and 57 (15.5%) from the labor ward. The reasons for the high number of newborns screened in the Neonatology unit and post partum wards include (1) availability of newborns in neonatology and postpartum wards. (2) Most newborns were discharged early (at the 6th hour) from the labor ward. (3) Most newborns screened to replace missing samples because of early discharge were from Neonatology units and postpartum wards.

We have used a larger sample size compared to studies in South Africa which screened 150 [8], 121 [10], and 121 newborns [17]. However, it was smaller compared to studies in Canada [5], France [13], USA [11, 18], India [14], South Africa [15], Netherlands [17], Brazil [16,19], Greece [21], and Belgium [23]. The reasons include (1) their study period was much longer (1–10 years) compared to our study period which was only two months. (2) Some included age groups of infants and children. (3) Most performed diagnostic audiologic work-up. (4) In many studies, more than one center and/or hospital (Colorado NHS used 26 hospitals) and cities were included in the study [10–15]. In general, our sample size was significantly larger compared to the studies conducted in single centers, similar setups, as well as similar age groups.

The results of this study show that referral rates were greater for newborns screened after 7 days of life, which is different from other studies [4, 5, 8]. The assumed reasons are most newborns older than 7 days were from the Neonatology unit. Many of those had more than one risk factor for hearing loss that could affect the screening outcome. Screening in the Neonatology unit has a high ambient noise which significantly affects the outcome (*P* value <0.001).

Based on a systemic review on the effects of the screening protocol, the referral rate of OAE screening ranges from 1.3% to 39.7% [5]. In our study, the overall refer rate of TEOAE was 17.5%, which is closer to the referral rate (10.5%) reported in Turkey [8]. However, it was significantly lower compared to the referral report of Nigeria (33.2%) and Brazil (30.0%). The overall refer rate of AABR was 88.5%, significantly higher compared to the reports of South Africa (16.7%), India (9.1%), Germany (3.8%), and Turkey (2%) [8].

A number of factors contribute to the high refer rate of AABR in our study. First, the test environment was neither dedicated nor in a soundproof room as we used available spaces such as Neonatology unit, labor ward, and postpartum wards. Secondly, most newborns screened with AABR were from Neonatology units with multiple risk factors (69 had one risk factor and 55 had two risk factors), noise of the environment, and electrical machines (mechanical ventilators and electrocautery), which interfere with AABR studies [1–3]. Thirdly, these newborns could have hearing loss, which needs follow-up and diagnostic audiologic work-up. Fourth, AABR was used only in the third stage of screening compared to other studies which used it in each stage. In general, our referral rate has decreased with the use of repeat OAE tests and the use of ABR, which is consistent with the findings of previous studies [1, 2, 4–6, 8].

In general, the overall refer rate of our study was 15.5%, which is similar to the refer rates of Ghana (16.9%) and Colombia (15.9%) but significantly lower than the refer rate of Nicaragua (30.8%) [16–23]. Nevertheless, it was significantly high compared to the referral rate of United Kingdom (2.6%), Italy (3.8%), Germany (5.5%), Belgium (2.8%), France (1.5%), USA (1.6%), and Brazil (0.2%) [24].

Newborns with very low birth weight and jaundice were significantly associated with the refer result. Avoidable factors, such as noise and vernix caseosa, were also found to be significantly associated with refer rates which is similar to the previous finding in infants [2, 8–10]. Most of our references studied associated factors with hearing loss since they performed diagnostic audiologic work-up. Therefore, it is difficult to compare with our study.

The proportion of newborns lost to follow-up (expressed as a percentage of the total number of newborns screened) also displayed a wide range, from 0% to 11% [5]. In our study, the proportion of lost to follow-up (expressed as a percentage of the total number of newborns screened) was 5.2%, which is in line with the above study.

In general, the outcome of this study shows 80.1% (295) passed, 14.8% (54) had refer results, and 5.1% [19] were lost to follow-up. The overall refer rate of TEOAE and ABR was 15.5% (54). 54 of the newborns were linked to the ENT department for audiologic diagnostic work-up.

## 5. Conclusions and Recommendations

In general, the magnitude of newborns with overall screening is 15.5%, and hence, out of 368 newborns, 54 of them need diagnostic audiologic work-up. Newborns with very low birth weight and neonatal jaundice (requiring exchange transfusion) had a statistically significant association with the refer results. Likewise, noise exposure and vernix caseosa were also found to be significantly associated with the refer result of the screening test, although they are avoidable factors. Clinicians involved in newborn screening should perform otoscopic examination before screening, removal of vernix caseosa, repeat screening, and perform screening in quite dedicated space.

We recommend screening of newborns with jaundice as well as very low birth weight. We recommend to our institution (SPHMMC) to develop NHS programs. We also recommend to health policymakers, the Ethiopian Ministry of Health, to consider NHS programs. We also recommend a larger community-based representative study with full diagnostic audiologic work-up since our study is single centered and may not show the full picture of hearing loss in newborns in our society.

Some of the challenges to implement NHS in this study were an early hospital discharge within 6 hours, a high false positive rate (20.5%), and newborns lost to follow-up (5.2%). High false positive rates can be avoided by repeated testing using two-or three-stage screening as well as diagnostic audiologic work-up. Lost to follow-up can be alleviated by combining NHS with other routine newborn or maternal health visits such as postpartum follow-up on the 6^th^ day, during circumcision, and/or immunization at 3 months. Training dedicated health professionals (midwifes and nurses) to create awareness of families about the importance of screening and its impact on newborns' development of speech, language, and cognitive activity and also the developing of a sufficient record system to call parents for follow-up. Other challenges include the lack of trained personnel to provide screening and insufficient amount of screening instruments.

### 5.1. Strength and Limitations of the Study

We have used the UNHS protocol which is recommended by most studies and guidelines. A two-stage screening test was done, which is the recommended one by WHO, JCIH, USPSTF, and the American Academy of Pediatrics for detection and intervention. During follow-up, we didn't do diagnostic audiologic work-up because of time and resource constraints. The irregular follow-up schedule of newborns starting from the second day till 30 days of age was used, as well as early discharge.

## Figures and Tables

**Table 1:** The refer rate of newborns according to gestational age and birth weight (5).

Factors		Number of newborns	Pass TEOAE	Refer TEOAE	Refer rate (%)
Prenatal and obstetric related factors	SVD	252	162	88	35
	C/S	103	51	52	50.5
	Vacuum	9	9		
	Forceps	4	4		
	Gestational age ≥34 weeks	338		118	35
	Gestational age >34 weeks	30	8	22	73.3
	Normal birth weight [2500–4000 gram]	262	175	87	33.20
	Low birth weight [1500–2500)	77	46	31	40.30
	Very low birth weight (<1500 gram)	29	7	22	75.90

Postnatal factors	NICU admission ≥5 days	49	21	28	57.1
	Aminoglycoside administration ≥5 days	69	27	42	60.1
	Neonatal jaundice	38	17	21	55.3
	Neonatal sepsis	55	24	31	56.4
	Perinatal asphyxia	10	3	7	70
	Meningitis/encephalitis	4	2	2	50

**Table 2:** Bivariate and multivariate regression analysis of factors associated with the first screening outcome of TEOAE (6).

First screening factors (TEOAE)	Bivariate regression analysis			Multivariate regression analysis		
	COR	95% CI	*P* value	AOR	95% CI	*P* value
>7 days–28 days			0.041	.	.	0.863
≤24 hours	0.530	0.289–0.973	0.041	1.100	0.422–2.867	0.845
>24 hrs–7 days	0.421	0.213–0.831	0.013	1.244	0.520–2.979	0.623
Gestational age ≥34 weeks	.			.	.	0.295
Gestational age <34 weeks	5.127	2.214–11.871	0.000	1.571	0.470–5.249	0.463
Normal birth weight [2500–4000 gram]	.		0.000	.	.	0.049
Very low birth weight <1500 gram	6.322	2.6–15.372	0.000	4.338	1.138–12.395	0.015
Noise	4.925	3.126–7.758	0.000	4.746	2.505–8.992	0.001
Vernix caseosa	8.231	4.329–15.650	0.000	19.745	9.057–43.043	0.001
Neonatal jaundice	2.910	1.112–4.314	0.023	0.964	0.396–2.348	0.936
NICU admission for ≥5 days	2.464	1.338–4.539	0.004	0.509	0.152–1.703	0.273
Neonatal sepsis	2.417	1.352–4.324	0.003	4.258	0.974–18.604	0.054
Perinatal asphyxia	3.947	1.004–15.525	0.049	4.073	0.905–18.342	0.067

**Table 3:** The refer rate of follow-up screening of TEOAE according to the mode of delivery, gestational age, and birth weight (6).

Factors	Total number of newborns	Frequency of pass TEOAE	Frequency of refer TEOAE	Refer rate (%)
C/S	44	20	24	54.50
SVD	74	38	36	48.60
Forceps	3	2	1	33.30
Gestational age ≥34 weeks	101	60	41	40.6
Gestational age <34 weeks	20		20	100
Normal birth weight [2500–4000 gram]	78	48	30	38.50
Low birth weight [1500–2500 gram)	22	10	12	54.50
Very low birth weight <1500 gram	21	2	19	90.50

**Table 4:** Bivariate and multivariate regression analyses of factors associated with follow-up screening outcomes of TEOAE—7.

First screening factors (TEOAE)	Bivariate regression analysis			Multivariate regression analysis		
	COR	95% CI	*P* value	AOR	95% CI	*P* value
Normal birth weight [2500–4000 gram]	.		0.002	.	.	0.495
Low birth weight [1500–2500 gram)	1.920	.739–4.990	0.181	0.326	0.049–2.176	0.247
Very low birth weight <1500 gram	15.200	3.302–69.969	0.001	0.421	0.031–5.768	0.517
Noise	51.409	11.433–231.174	0.001	39.445	5.974–260.467	0.001
Neonatal jaundice	26.690	3.438–207.213	0.002	21.633	1.540–303.994	0.023
NICU admission for ≥5 days	38.270	4.965–294.961	0.001	1.643	0.051–52.510	0.779
Aminoglycoside administration ≥5 days	34.179	7.650–152.696	0.001	15.521	0.722–333.488	0.080

## Data Availability

The original data used to support the findings of this study were supplied by the institution under license and so cannot be made freely available. The requests for access to these data should be made to Werkineh/Haben Birhane, (havenasvan@yahoo.com).
